# Energy balance and the sphingosine-1-phosphate/ceramide axis

**DOI:** 10.18632/aging.101347

**Published:** 2017-12-13

**Authors:** Cara Green, Sharon Mitchell, John Speakman

**Affiliations:** Institute of Biological and Environmental Sciences, University of Aberdeen, Aberdeen, Scotland, UK

**Keywords:** sphingosine-1-phosphate, ceramide, calorie restriction, energy balance, hunger

The bioactive signalling lipid sphingosine-1-phosphate (S1P) was discovered over 25 years ago as a regulator of cell proliferation. Since that time, S1P has been implicated across multiple cellular signalling systems, including cell survival, migration and differentiation. As a result, S1P has been associated with several health conditions and non-communicable diseases including cancer, inflammatory disorders, diabetes and atherosclerosis, much of which is attributed to its role in immune cell trafficking. S1P exerts its cellular effects through G-protein coupled receptors: S1P receptors 1-5 (S1PR1-5) [1].

Levels of cellular S1P are modulated through the deacylation and phosphorylation of ceramide, the interconversion and subsequent ratio of these molecules is referred to as the S1P/ceramide axis. Many of the cellular signalling systems modulated by S1P are conversely affected by ceramide for example S1P promotes growth and inhibits apoptosis whereas ceramide has the opposite effects on these processes [2].

More recently, an emerging role for the S1P/ceramide axis in the regulation of energy balance has been indicated in several studies. In 2014, Silva et al. demonstrated that S1P receptor 1 (S1PR1) was highly enriched in pro-opiomelanocortin (POMC) neurons in the hypothalamus of rats. The hypothalamus is a key sensor and regulator of nutrient signals [3] and POMC neurons in particular are involved in leptin signal transduction pathway and are thought to help signal satiety and inhibit feeding (anorexigenic). Silva et al. also found that S1P had an anorectic effect of rats, as intracerebroventricular (ICV) injections of S1P reduced food consumption and increased energy expenditure. The study also indicated that these changes were mediated through leptin signal transduction as S1P injection resulted in a dose-dependent increase in tyrosine phosphorylation of both Janus kinase 2 (Jak2) and signal transducer and activator of transcription 3 (STAT3), signalling of which controls anorexigenic and thermogenic signalling in the hypothalamus. In addition, disruption of S1PR1 in the arcuate nucleus caused hyperphagia, low levels of STAT3 phosphorylation and reduced energy expenditure [4]. Further studies by the same group indicated that S1P is increased by exercise in the cerebrospinal fluid (CSF) of young rats, and transferring CSF from these rats into the hypothalamus of middle-aged rats reduced food intake.

In addition, chronic exercise increased both S1PR1 and STAT3 phosphorylation in the hypothalamus of middle aged mice, mirroring previous effects seen upon ICV injection of S1P in rats. ICV injection of S1PR1 activators also induced STAT3 phosphorylation in the hypothalamus and reduced food intake in middle aged rats [5].

Da Silva also found that a high fat diet (HFD) resulting in obesity not only caused hypothalamic leptin resistance but also decreased S1PR1 protein levels in both mice and rats [4]. This effect however has been shown to be reversible in male C57B/6J mice fed on a HFD that were injected intraperitoneally with S1P analogue FTY720, it blunted accumulation of large adipocytes, promoted lipolysis and inhibited adipogenesis. FTY720 also decreased lipid accumulation in maturing preadipocytes and down-regulated markers of adipogenic differentiation. FTY720 also induces protein kinase B (Akt) phosphorylation, which is known to improve insulin sensitivity and reduce fat accumulation [6].

**Figure 1:**
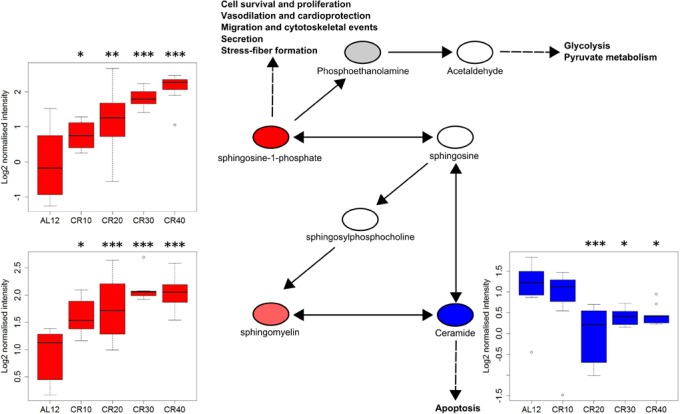
Changes in the S1P and ceramide biosynthesis pathway with increasing CR Figure modified from IPA, boxplots showing outliers, minimum, maximum and interquartile range values. Red=increased with CR, blue = decreased with CR, white = undetected in our analysis. Metabolite intensities log2 transformed. ^*^=P<0.05, ^**^=P<0.01, ^***^=P<0.001. Figure taken from Green et al., 2017 [7].

Conversely, calorie restriction (CR) has also been shown to alter S1P levels, in our recent study mice undergoing calorie restriction (CR) showed a hepatic increase in S1P and sphingomyelin whilst ceramide was decreased. Furthermore, this response was dose dependent, with the response becoming more pronounced with increasing CR (Figure 1). Similar to the effects seen in obese mice injected with S1P analogue FTY720, CR also produces a metabolic shift from lipogenesis to lipolysis, which may in part be attributed to changes in the S1P/ceramide axis [6,7]. In our study we found that S1P correlated negatively with body temperature and positively with food anticipatory activity, indicating that it may also have a role in the physical and behavioural changes associated with hunger [7]. However, intraperitoneal injections of S1P and an S1PR1 agonist were not effective in changing food intake or body temperature [7]. Unlike in the previous studies where direct ICV injection of S1P produced a response through the JAK/STAT pathway, our findings suggest that S1P may only act locally [4,5]. However, we only tested a limited dose range so this requires further investigation.

Taken together, these findings suggest a significant role of the S1P/S1PR1 axis in the hypothalamus, adipose tissue and liver. In particular activation of hunger signalling pathways through S1P produces an anti-obesity effect in mice and rats, through increasing energy expenditure, increasing lipolysis, decreasing lipogenesis and food intake. The S1P/S1PR1/ceramide axis, in addition to downstream signalling pathways, seems to be inducible by both exercise and CR. These studies provide insight into how S1P analogues may be used as a therapeutic agent in the treatment of obesity, and the importance of further work to understand the organ specific effects of S1P.
